# Effects of an Animal-Derived Biostimulant on the Growth and Physiological Parameters of Potted Snapdragon (*Antirrhinum majus* L.)

**DOI:** 10.3389/fpls.2018.00861

**Published:** 2018-06-20

**Authors:** Giuseppe Cristiano, Emanuele Pallozzi, Giulia Conversa, Vincenzo Tufarelli, Barbara De Lucia

**Affiliations:** ^1^Department of Agricultural and Environmental Sciences, University of Bari Aldo Moro, Bari, Italy; ^2^Institute of Agro-Environmental & Forest Biology, National Research Council (CNR), Rome, Italy; ^3^Department of the Science of Agriculture, Food and Environment, University of Foggia, Foggia, Italy; ^4^Section of Veterinary Science and Animal Production, Department of DETO, University of Bari Aldo Moro, Bari, Italy

**Keywords:** biomass, gas exchange, protein hydrolysates, root architecture, sustainable production

## Abstract

To assess the effect a new animal-derived biostimulant on the growth, root morphology, nitrogen content, leaf gas exchange of greenhouse potted snapdragon, three treatments were compared: (a) three doses of biostimulant (D): 0 (D_0_ or control), 0.1 (D_0.1_), and 0.2 g L^−1^ (D_0.2_); (b) two biostimulant application methods (M): foliar spray and root drenching; (c) two F_1_ *Antirrhinum majus* L. hybrids (CV): “Yellow floral showers” and “Red sonnet.” The treatments were arranged in a randomized complete-block design with four replicates, with a total of 48 experimental units. Plant height (+11%), number of shoots (+20%), total shoot length (+10%), number of leaves (+33%), total leaf area (+29%), and number of flowers (+59%) and total aboveground dry weight (+13%) were significantly increased by the biostimulant application compared to the control, regardless of the dose. The lowest dose resulted in the best effect on the ground plant dry weight (+38%) and, in order to the root system, on total length (+55%), average diameter (+36%), volume (+66%), tips (+49%), crossings (+88%), forks (+68%), projected (+62%), and total surface area (+28%). Compared to the control, plants treated with the biostimulant significantly enhanced leaf (+16%) and root (+8%) nitrogen content, photosynthetic rate (+52%), transpiration rate (+55%), and stomatal conductance (+81%), although there were no changes in dark-adapted chlorophyll fluorescence. Differences in the application method were not evident in the aboveground morphological traits, except in the plant shoot number (root drenching: +10%). The foliar spray compared to root drenching had a significant effect only on flower dry weight (3.8 vs. 3.0 g plant^−1^). On the other hand, root drenching had a positive effect on ground dry weight (2.7 vs. 2.3 g plant^−1^), root morphology, leaf-N and root-N content (+3%), transpiration rate (+21%), stomatal conductance (+40%), concentration of CO_2_ in intracellular spaces (+11%), as well as on the efficiency of Photosystem II (+11%). A higher pot quality was obtained in “Red sonnet” compared to “Yellow floral shower.” Based on our findings, applying the biostimulant to potted snapdragon at the lowest dose, as part of a fertilizing regime, improves the crop quality in an agro-environmental sustainable way.

## Introduction

Biostimulants are environmental-friendly substances that can increase crop yield by acting on plant metabolism (Yakhin et al., 2017), thus improving nutrient use efficiency (Vernieri et al., 2006; De Pascale et al., 2017) and affecting both root growth (Zeljkovic et al., 2010; De Lucia and Vecchietti, 2012) and root architecture (Yazdani et al., 2014). They can have both a direct or indirect effect on plants. They can alter the biological, biochemical, and physical properties of the soil (Rouphael et al., 2017a,c), enhance the performance of plants under abiotic stress (Van Oosten et al., 2017) and they can also impact on the overall transcriptome profile by modifying the plant metabolome (Battacharyya et al., 2015). Biostimulants are used with root drenching or foliar spray application (Kunicki et al., 2010), in addition to fertilizers to boost their action (Mugnai et al., 2008).

Biostimulants are composed of bioactive compounds (Calvo et al., 2014; Du Jardin, 2015) such as amino acids, peptides, humic substances, seaweed extracts, and other beneficial elements (Colla et al., 2014; Nardi et al., 2016). Protein hydrolysates (PHs) are an important group of biostimulants, with a high content of peptides and amino acids, and therefore they display a positive effect on crop performance (Colla et al., 2017). Both PHs from animal- and plant-derived raw materials (Colla et al., 2015), act when applied at low rates (Zhang et al., 2003; Kauffman et al., 2007; Kunicki et al., 2010; Ertani et al., 2016).

Compared to plant PH biostimulants, animal PH biostimulants have a higher nitrogen content ranging from 9 to 16% of total dry matter, and they are released more gradually (Polo et al., 2006). Polo and Mata (2018), evaluating the effects of different doses of an enzymatically hydrolyzed PH biostimulant (Pepton) compared to a seaweed biostimulant (Acadian) on cherry tomatoes, showed that both biostimulants provide amino acids (much more in case of Pepton) and minerals (mainly iron in case of Pepton and sulfur and boron in case of Acadian) that enhanced growth and yield. Glycine and proline are the most abundant amino acids in collagen-based biostimulants, while glutamic acid is dominant in vegetal-based biostimulants (Baroccio et al., 2017). The production process is extremely important in determining the final PHs composition too: chemical hydrolysis lowers the tryptophan content; on the other hand enzymatic hydrolysis, combined with a temperature of 60°C, reduces such amino acid losses (Tuomisto and Teixeira de Mattos, 2011). A few cases of phytotoxicity and plant growth depression have been found after using commercial animal PH biostimulants, which could be due to incorrect product concentration and/or sub-optimal field conditions (Ruiz et al., 2000; Cerdán et al., 2009; Lisiecka et al., 2011). The correct application of animal PH biostimulants could be a way of both decreasing the use of mineral fertilizers and reducing the disposal of animal-processing wastes.

Bulgari et al. (2015) reported that biostimulants enhance both vegetative and blooming performance in the greenhouse cultivation of bedding plants. Among ornamental bedding plants, snapdragon (*Antirrhinum majus* L., *Plantaginaceae* family), known also for its medical properties (Bulír, 2009), is one of the most important floricultural plants used as cut flowers, pot plants and landscaping purposes (Carter and Grieve, 2008; Asrar et al., 2012). Thus, the fine tuning of agronomical protocols aimed to improve both the plant growth and quality is of great interest in the cultivation of snapdragon.

To the best our knowledge, the use of PH-based biostimulants in ornamental potted plant production is still poorly studied, despite their important contribution to the sustainability of ornamental production.

The aim of this research was to assess the effects of animal-derived PH biostimulant on the growth and blooming parameters, nitrogen plant content, root morphology, leaf gas exchange, and chlorophyll fluorescence in greenhouse potted snapdragon plants.

## Materials and methods

### Experimental conditions

The experiment was carried out from 1 December 2015 to 14 May 2016 (166 days), in a heated greenhouse, covered with ethyl vinyl acetate (EVA) plastic film, located in Terlizzi (Bari, Southern Italy, 41° 07′ 55″ N, 16° 32′ 45″ E, 180 m a.s.l.), and equipped with environmental control software (Clima control/Pro, Ragusa, Italy). Natural photoperiod, mean air temperature of 20/13°C day/night, and 65% relative humidity inside the greenhouse were maintained throughout the growth stages.

### Treatments and experimental design

Three treatments were compared: (a) three doses of biostimulant (D): 0 (D_0_ or control), 0.1 (D_0.1_), and 0.2 g L^−1^ (D_0.2_); (b) two biostimulant application methods (M): foliar spray and root drenching; (c) two F_1_ *Antirrhinum majus* L. hybrids (CV): “Yellow floral showers” (Sakata seeds) and “Red sonnet” (Sakata seeds). The two hybrids, both ideal for spring production, are characterized by a different growth behavior: the first is a *nanum pumilum* with a dwarf habit, while the second has a sturdy branching.

Thirty-day-old healthy and uniform size seedlings with three pairs of leaves were produced in plug trays by a specialized nursery. On 1 December 2015 the seedlings were individually transplanted into 2.0 L plastic pots filled with a mixture of potting substrate (Plantaflor®, Germany) and perlite (4:1 v/v). The final substrate mixture was healthy and well drained. It had a pH of 5.7 and an EC of 1.5 dS m^−1^. Pots were arranged on the ground, covered with mulching film, at a density of 15 plants m^−2^.

Each experimental unit consisted of eight plants. The treatments were arranged in a randomized complete-block design with four replicates, with a total of 48 experimental units. The biostimulant used was an animal-derived PH product (Hydrostim®, Hydrofert, Italy) obtained through the enzymatic hydrolysis of proteins from erythrocytes (red blood cells) under alkaline conditions (enzymatic kit with producer details), containing 38% organic matter, 10.2% total nitrogen, and 52% amino acids and soluble peptides (Table 1).

**Table 1:** Amino acid content of the animal protein hydrolysates used as a biostimulant on snapdragon plants (“Yellow floral shower” and “Red sonnet”).

**Amino acid**	**Content (mg L^−1^)**	**Amino acid**	**Content (mg L^−1^)**
Glycine	10.90	Serine	1.62
Glutamic acid	6.52	Phenylalanine	1.24
Proline	6.50	Isoleucine	0.86
Hydroxyproline	5.28	Cysteine + Cystine	0.46
Alanine	4.94	Methionine	0.38
Aspartic acid	3.45	Tyrosine	0.34
Arginine	2.98	Threonine	0.34
Leucine	2.21	Valine	0.15
Betaine	2.02	Histidine	<DL
Lysine	1.85	Tryptophan	<DL

*DL, detection limit*.

Biostimulant treatments started 45 days after transplanting (on 14 January) and were applied weekly eight times, until flower bud differentiation (on 3 March).

Foliar spray treatments were applied on the leaves of snapdragon plants at the dose of 150 mL/plant using a hand sprayer. Care was taken to ensure no dripping occurred onto the substrate. Root drenching treatments were performed with the same volume (150 mL/plant) which was applied directly on top of the growing media. The same volume of tap water was applied to the foliar spray and root drenching control plants.

Plants were fertigated starting 1 month after transplantation by a micro-irrigation system with a nutrient solution containing 40 mg L^−1^ N, 8 mg L^−1^ P, 60 mg L^−1^ K, 44 mgL^−1^ Ca, and 8 mg L^−1^ Mg, plus microelements (Fe: 3 mg L^−1^, Mn: 2 mg L^−1^, Cu: 0.1 mg L^−1^, Bo: 0.5 mg L^−1^) with an electrical conductivity (EC) of 1.2 dS m^−1^ at 25°C and with a pH 6.0 ± 0.1. Apart from the fertilization, cultivation was conducted following the grower's standard practices.

### Growth measurements, root morphology, and ornamental characteristics

At the end of the cultivation period (166 days after transplantation), the growing medium was gently washed from the roots, and the plants were divided into shoots, leaves, flowers, and roots. These were then oven dried at 70°C until they reached a constant dry weight.

Five plants per treatment were harvested and their height, total shoot length, number of shoots, leaves, and flowers were measured. Total leaf area per plant was also determined by a leaf area meter (Delta-T, Decagon Devices, Pullman, Washington, USA). Total above-ground (shoot+leaves+flowers) and ground fresh and dry weight were calculated.

Fresh root systems were carefully washed with tap water after harvest, spread out on a transparent tray, and scanned at 400 dpi with a scanner (Epson Expression © 10000 XL, Japan). The captured images were then processed using image analysis software (WinRHIZO v. 2005b ©, Regent Instruments Inc., Québec, Canada) to determine total root length, average diameter, volume, tips, forks, crossings, projected, and surface area. For each replicate and treatment, roots of three plants were scanned.

Total leaf and root nitrogen content was measured using 1 g samples of foliar and root tissues, using the Kjeldahl method after 96% H_2_SO_4_ hot digestion.

### Gas exchange and chlorophyll fluorescence measurements

At the phenological stage of full flowering of plants, leaf gas exchange was measured using an IRGA (LI-6400XT portable gas exchange system, Li-COR, Lincoln, NE, USA), equipped with a 2 cm^2^ leaf chamber with a built-in fluorescence system (LI-6400-40, Li-COR, Lincoln, NE, USA).

The chamber air flow and CO_2_ concentration were set at 300 μmol s^−1^ and 400 ppm, respectively. Measurements were performed at the same time of the day (9:00–11:00 and 13:00–15:00 p.m. CET Time) to minimize the physiological changes driven by environmental factors on fully expanded mature leaves of the same age. The fluorescence measurements were performed on the plants with a different order each day and no shift in parameters was noted during the day as we avoided the early and late hours. The plants were never under water stress.

Leaves were exposed to a saturating photosynthetic photon flux density of 1000 μmol m^−2^ s^−1^, at a temperature of 25°C and with the relative humidity within the leaf cuvette ranging between 40 and 60%. The parameters were recorded when the leaves inside the chamber reached a steady-state status. The instrument provides a continuous display of gas exchange parameters. Steady-state was reached when the first decimal number of photosynthesis was stable (and therefore the other parameters). This usually happened after 2–3 min, The internal chamber fan was set to maximum speed producing a fast air turnover inside the small fluorescence chamber.

Photosynthesis (A), stomatal conductance (g_s_), and internal concentration of CO_2_ (C_i_) were calculated by Li-COR software. The electron transport rate (ETR), maximum quantum efficiency of PSII (F_v_/F_m_) and the actual quantum yield of PSII in illuminated leaves (F'_v_/F'_m_) were measured following a saturating pulse of light (10,000 μmol m^−2^ s^−1^). The gas exchanges and fluorescence data presented are means from at least eight leaves per replication. F_v_/F_m_ determinations were performed after adapting the leaves to the dark for 30 min. Shading clips were used on the measured leaves, and the plant to be measured was also placed in a dark room.

### Statistical analysis

The data were analyzed by three-way ANOVA using CoStat—Statistics Software. Treatment means were separated with Duncan's multiple range test (*P* ≤ 0.05).

## Results

### Morphological and qualitative traits

The main effects of the biostimulant dose, application method and cultivar on morphological characteristics are reported in Table 2. The snapdragon height was significantly increased by the biostimulant application compared to untreated plants (+11%), A similar trend was observed in terms of the number of shoots per plant (+20%), total shoot length (+10%), number of leaves per plant (+33%), total leaf area (+29%), and number of flowers per plant (+59%). No significant differences were found by comparing the effects of the two doses of biostimulant. The root application method only had an effect on the plant shoot number (+10%). The cultivar factor also had a significant effect on all morphological characteristics. However, data analysis showed a significant interaction between the application method and cultivar regarding total shoots length and leaves per plant. The interaction between the dose and cultivar was also significant in terms of the number of shoots, leaves, and flowers per plant. The interaction-effect between the dose and application method was significant only in terms of the number of both shoots and flowers per plant (Table S1, Supplementary materials), whereas the interaction dose ^*^ application method ^*^ cultivar was found to be significant for total shoot length and total leaf area (Table S2, Supplementary materials).

**Table 2:** Main effects of biostimulant dose, application method and cultivar on plant height, shoot number, total shoot length, leaves number, total leaf area, and flowers number in snapdragon plants.

**Treatments**	**Plant height (cm)**	**Shoots (n/plant)**	**Total shoot length (cm /plant)**	**Leaves (n/plant)**	**Total leaf area (cm^2^ /plant)**	**Flowers (n/plant)**
**DOSE (g L^−1^) (D)**						
0	33b	5.3b	168.7b	500a	619b	59b
0.1	36a	6.5a	183.3a	670a	789a	88a
0.2	37a	6.2a	190.6a	656a	805a	100a
**METHOD (M)**						
Foliar spray	36a	5.7b	183.0a	592a	784a	86
Root drenching	35a	6.3a	178.4a	626a	755a	80a
**CULTIVAR (CV)**						
Yellow floral showers	24b	5.3b	95.6b	416b	496b	53b
Red sonnet	48a	6.7a	266.1a	802a	1044a	112a
**SIGNIFICANCE**						
D	^*^	^**^	^*^	^***^	^*^	^***^
M	ns	^*^	ns	^*^	ns	^*^
CV	^**^	^*^	^**^	^**^	^***^	^**^
D × M	ns	^*^	ns	ns	ns	^*^
D × CV	ns	^**^	ns	^*^	ns	^*^
M × CV	ns	ns	^**^	^**^	ns	ns
D × M × CV	ns	ns	^*^	ns	^**^	ns

*Different letters within each column indicate significant differences according to Duncan's multiple-range test (P ≤ 0.05)*.

*ns, non significant*,

* *P < 0.05*,

** 0.01, and

*** *0.001, indicate level of significance*.

### Plant biomass

In the present experiment, snapdragons grown under the biostimulant treatment had a greater content of dry weight in terms of the different above-ground plant parts (shoots, leaves, and flowers) in comparison to the control (Table 3). The lowest dose increased the shoot dry weight (+11%) compared to the control, both leaf and flower dry weights were significantly increased by the biostimulant application (respectively +16 and 41%).

**Table 3:** Main effects of biostimulant dose, application method and cultivar on dry weights in various parts of snapdragon plants.

**Treatments**	**Dry weight (g/ plant)**				
	**Above ground parts**				**Underground parts**
	**Shoot**	**Leaf**	**Flower**	**Total**	
**DOSE (g L^−1^) (D)**					
0	22.7c	8.0b	2.7b	33.4b	2.1c
0.1	25.3a	9.2a	3.8a	38.4 a	2.9a
0.2	23.6b	9.4a	3.7a	36.7a	2.5b
**METHOD (M)**					
Foliar spray	23.9a	8.7a	3.8a	36.4 a	2.3b
Root drenching	23.9a	9.1a	3.0b	35.9 a	2.7a
**CULTIVAR (CV)**					
Yellow floral showers	15.8b	6.3b	2.3b	24.4 b	2.0b
Red sonnet	32.0a	11.4a	4.5a	48.1 a	3.1a
**SIGNIFICANCE**					
D	^*^	^**^	^**^	^**^	^***^
M	ns	ns	^***^	ns	^***^
CV	^***^	^**^	^**^	^**^	^**^
D × M	ns	ns	^***^	ns	^**^
D × CV	ns	ns	^*^	^***^	^*^
M × CV	ns	ns	^***^	ns	^*^
D × M × CV	ns	ns	^**^	^*^	ns

*Different letters within each column indicate significant differences according to Duncan's multiple-range test (P ≤ 0.05)*.

*ns, non significant*,

* *P < 0.05*,

** 0.01, and

*** *0.001, indicate level of significance*.

Applying biostimulant as a foliar method significantly increased the dry weight of the snapdragon flowers compared to the root application method (+27%). The differences between cultivars were highly significant for all the three plant parts, where “Red sonnet” was characterized by the greater dry weight (shoots +102%, leaves +81%, and flowers +96%) than “Yellow flower showers.”

Plant total aboveground dry weight was significantly higher in both doses compared to the control: from 0 to 0.1 g L^−1^ dose dry weight increased by 15%, whereas from 0 to 0.2 g L^−1^ by 10%.

Applying the biostimulant at the lowest dose resulted in the best effect on the ground plant dry weight (+38%) compared to control plants. The application method had a highly significant effect only on ground plant dry weight (root vs. foliar application: +17%), whereas the cultivar factor greatly affected both above-ground and ground dry weights, where “Red sonnet” recorded the highest values, 48.1 and 3.1 g, respectively. Examining the different interactions among factors, all interactions were significant for ground dry weight (Tables S1, S3, S4, Supplementary materials); conversely, the only highly significant interaction was detected between the dose and cultivar for above-ground plant dry weight (Table S2, Supplementary materials).

The interactions M ^*^ CV were highly significant only for flower dry weight (Table 3), the same was found for the interaction between dose and application method (Table S1, Supplementary materials).

### Root morphology

**Figure 1:**
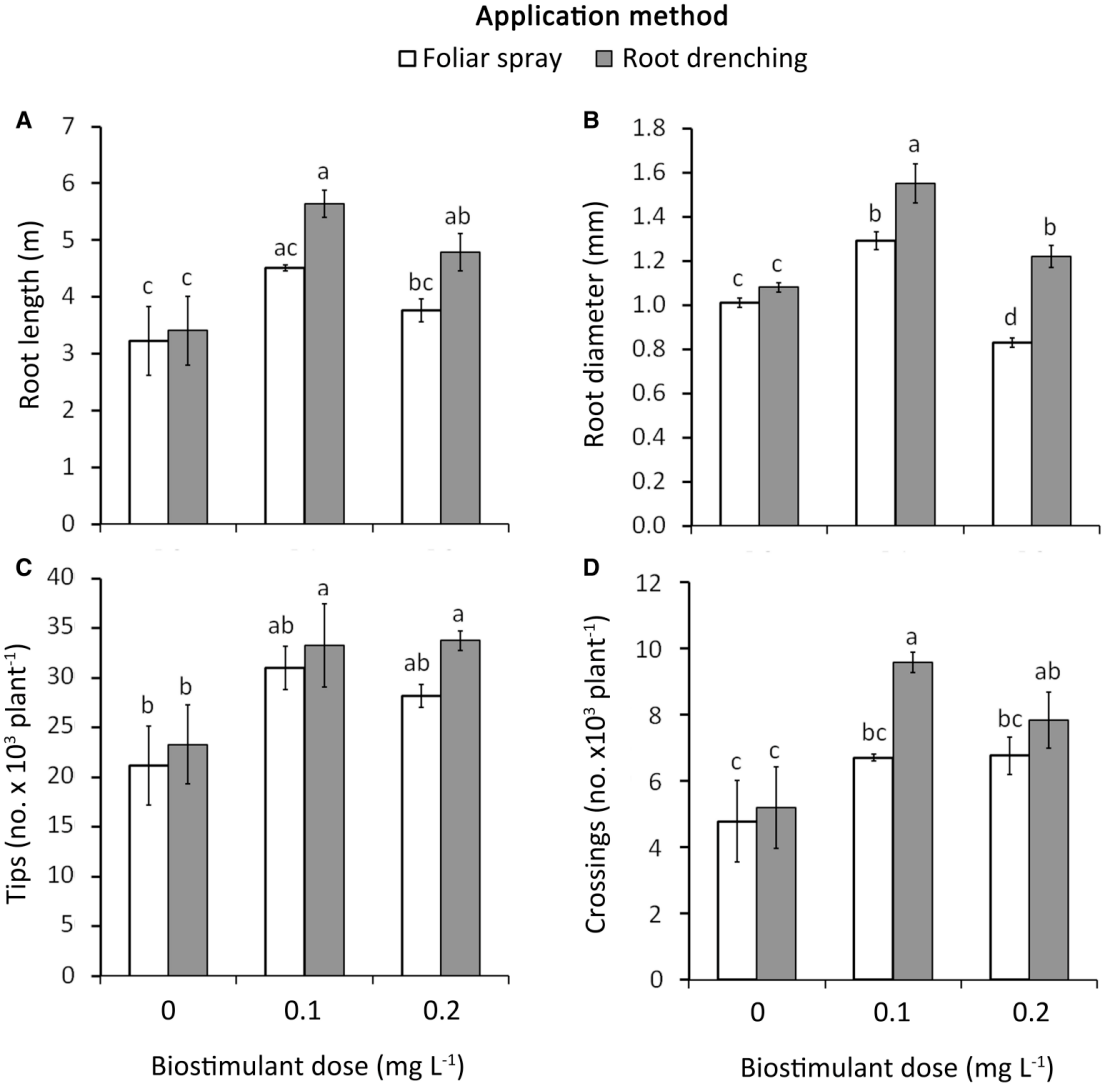
Effect of biostimulant dose (D_0_, D_0.1_, and D_0.2)_ and application method (F, foliar spray; R, root drenching) on root length **(A)**, root diameter **(B)**, tip number **(C)**, and crossing number **(D)** at the end of the experiment (166 DAT). Vertical bars (standard error) (*n* = 6) with different letters are significantly different according to Duncan's test (*P* = 0.05).

**Figure 2:**
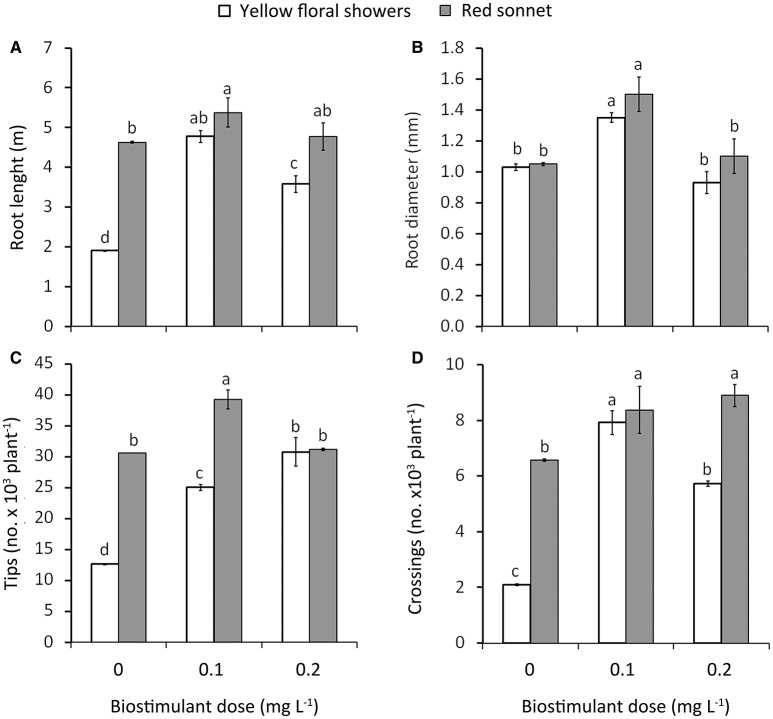
Effect of biostimulant dose (D_0_, D_0.1_, and D_0.2_) and cultivar (“Yellow floral shower” and “Red sonnet”) on root length **(A)**, root diameter **(B)**, tip number **(C)**, and crossing number **(D)** at the end of the experiment (166 DAT). Vertical bars (standard error) (*n* = 6) with different letters are significantly different according to Duncan's test (*P* = 0.05).

**Figure 3:**
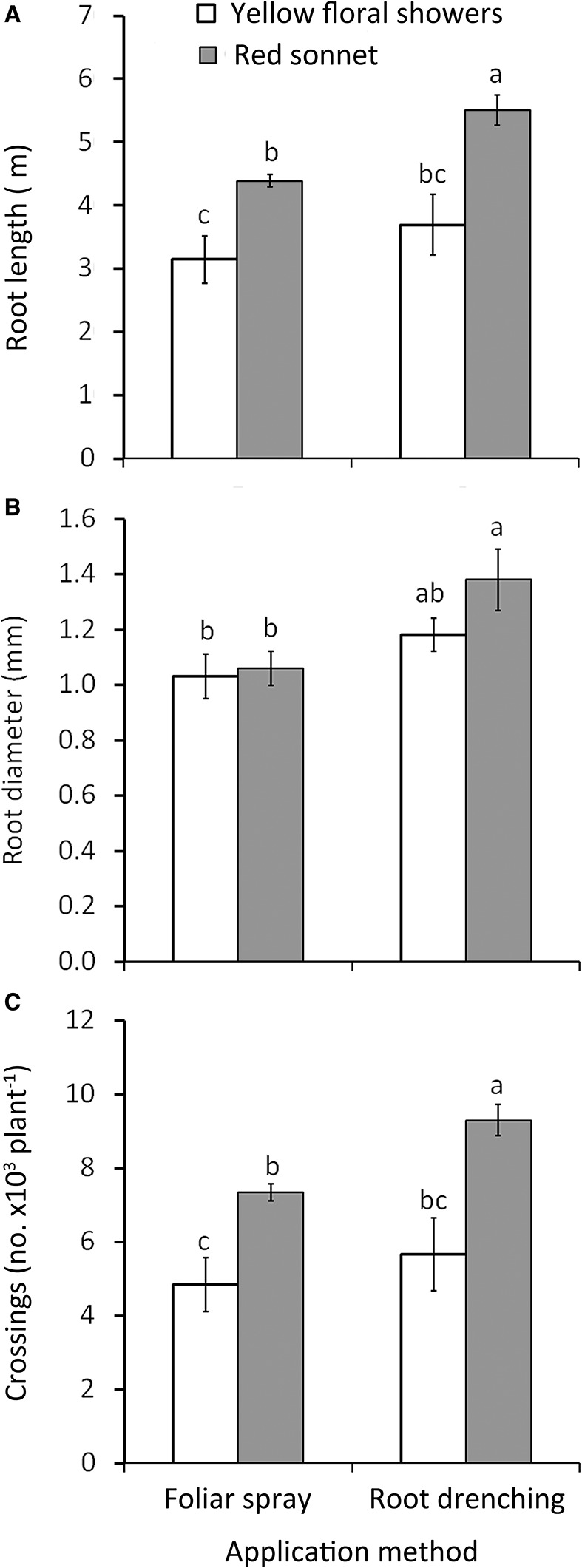
Effect of biostimulant application method (F, foliar spray; R, root drenching) and cultivar (“Yellow floral shower” and “Red sonnet”) on root length **(A)**, root diameter **(B)**, and crossing number **(C)** at the end of the experiment (166 DAT). Vertical bars (standard error) (*n* = 9) with different letters are significantly different according to Duncan's test (*P* = 0.05).

The application of an animal-derived PH biostimulant to snapdragon plants positively influenced the root morphology compared to the untreated plants (Tables 4, 5, Figures 1–3). Applying biostimulant at the dose of 0.1 g L^−1^ resulted in a significant improvement in total root length, average root diameter, and root volume in comparison to control plants, by 55, 36, and 66%, respectively. These significant increases were also seen in terms of root tips, crossings, and forks per plant, with the 0.1 g L^−1^ dose increasing the number of tips by 49%, crossings by 88%, and forks 68%.

**Table 4:** Main effects of biostimulant dose, application method and cultivar on total root length, root diameter, root volume, root tips, root crossings, and root forks in snapdragon plants.

**Treatments**	**Total root length (m 10^3^/plant)**	**Root diameter (mm)**	**Root volume (cm^3^/plant)**	**Root tips (n 10^3^/plant)**	**Root crossings (n 10^3^/plant)**	**Root forks (n 10^3^/plant)**
**DOSE (g L^−1^) (D)**						
0	3.3c	1.04b	4.60c	21.6c	4.3c	28.3c
0.1	5.1a	1.42a	7.65a	32.1a	8.1a	47.5a
0.2	4.2b	1.02b	5.66b	30.9b	7.3b	41.3b
**METHOD (M)**						
Foliar spray	3.8b	1.04b	5.29b	26.8b	5.9b	35.0b
Root drenching	4.6a	1.28a	6.65a	29.4a	7.2a	43.0a
**CULTIVAR (CV)**						
Yellow floral showers	3.4b	1.11b	5.15b	22.8b	5.2b	31.4b
Red sonnet	4.9a	1.21a	6.79a	33.6a	7.9a	46.7a
**SIGNIFICANCE**						
D	^**^	^**^	^**^	^**^	^**^	^**^
M	^**^	^*^	^*^	^*^	^*^	^*^
CV	^**^	^**^	^**^	^**^	^**^	^**^
D × M	^**^	^**^	^**^	^**^	^**^	^**^
D × CV	^**^	^*^	^**^	^**^	^*^	^*^
M × CV	^**^	^**^	^*^	ns	^**^	^**^
D × M × CV	^**^	^**^	^*^	^*^	^**^	^**^

*Different letters within each column indicate significant differences according to Duncan's multiple-range test (P ≤ 0.05)*.

*ns, non significant*,

* P < 0.05 and

** *0.01 indicate level of significance*.

**Table 5:** Main and interaction effects of biostimulant dose, application method and cultivar for projected and total root surface area in snapdragon plants.

**Treatments**	**Projected root area (cm^2^ /plant)**	**Total root surface area (cm^2^ /plant)**
**DOSE (g L^−1^) (D)**		
0	136.4c	414.2c
0.1	221.0a	697.6a
0.2	172.3b	545.3b
**METHOD (M)**		
Foliar spray	157.9b	493.0b
Root drenching	195.3a	611.7a
**CULTIVAR (CV)**		
Yellow Floral Showers	148.2a	468.0b
Red Sonnet	204.9b	636.7a
**D × M**		
D0 × Foliar spray	132.5d	401.9c
D0 × Root drenching	140.3d	426.5c
D0.1 × Foliar spray	193.4bc	611.1b
D0.1 × Root drenching	248.7a	784.2a
D0.2 × Foliar spray	147.9cd	466.1c
D0.2 × Root drenching	196.8b	624.6b
**D × CV**		
D0 × Yellow floral showers	95.7d	305.5c
D0 × Red sonnet	177.1b	522.9b
D0.1 × Yellow floral showers	210.8ab	665.6a
D0.1 × Red sonnet	231.3a	729.7a
D0.2 × Yellow floral showers	138.2c	433.1b
D0.2 × Red sonnet	206.5ab	657.6a
**M × CV**		
Foliar spray × Yellow floral showers	137.9b	432.7b
Root drenching × Red sonnet	158.4b	553.3b
Foliar spray × Yellow floral showers	177.4b	503.3b
Root drenching × Red sonnet	232.1a	720.1a
**SIGNIFICANCE**		
D	^***^	^***^
M	^***^	^***^
CV	^***^	^***^
D × M	^**^	^***^
D × CV	^***^	^***^
M × CV	^**^	^***^
D × M × CV	ns	^***^

*Different letters within each column indicate significant differences according to Duncan's multiple-range test (P ≤ 0.05). ns, non significant*,

** P < 0.01 and

*** *0.001, indicate level of significance*.

Using biostimulants as a drench method significantly enhanced the plant root morphology compared to foliar method; in addition, the “Red sonnet” resulted in the best root system response compared to “Yellow floral showers.” The interactions among factors were significant for all the plant traits related to the root morphology, except for root tips in Method ^*^ Cultivar (Table 4). Regarding the Dose ^*^ Method interaction, plants grown in both 0.1 and 0.2 g L^−1^ and treated with the drenching method, resulted in the highest values in root length (D_0.1_: 5.6 m, D_0.2_: 4.8 m; Figure 1A), root tip number (D_0.1_: 33.4 10^3^, D_0.2_: 33.7 10^3^; Figure 1C), and crossing number (D_0.1_: 9.6 10^3^, D_0.2_: 7.8 10^3^; Figure 1D). Conversely, for the root average diameter, the best value was recorded only in 0.1 g L^−1^^*^ Root drenching interaction (1.55 mm, Figure 1B).

Significant interactions were detected between biostimulant Dose ^*^ Cultivar (Figure 2): highest length values were recorded in plants treated with 0.1 g L^−1^^*^ “Red sonnet” (5.4 m, Figure 2A); in addition both 0.1 g L^−1^^*^ “Yellow floral showers” and 0.1 g L^−1^^*^ “Red sonnet” had the highest values in root average diameter (respectively 1.35 and 1.50 mm, Figure 2B), for tips: 0.1 g L^−1*^ “Red sonnet” with 39.2 10^3^ (Figure 2C). Crossing number resulted in the highest values in 0.1 g L^−1^, irrespective of the cultivars, and in 0.2 g L^−1*^ “Red sonnet” (Figure 2D). Between the Method ^*^ Cultivar interaction, the same significant trend was found in root drenching ^*^ “Red sonnet,” in terms of root length (Figure 3A), root' diameter (Figure 3B), and crossing number (Figure 3C).

Table 5 shows that a 0.1 g L^−1^ dose of biostimulant resulted in the best effect on both the projected and total surface root area, compared to the other treatments. The root drenching method significantly increased these traits compared to the foliar spray (respectively 195.3 vs. 157.9 cm^2^ and 636.7 vs. 468 cm^2^); “Red sonnet” responded more efficiently than “Yellow floral showers.”

Significant D ^*^ M, D ^*^ CV, and M ^*^ CV interactions were found for the projected and surface root area. 0.1 g L^−1*^ root drenching plants produced the highest values of both projected and total surface root area (248.7 and 784.2 cm^2^ respectively). In both cultivars, 0.1 g L^−1^ treatment showed the highest projected and total surface root area values; the same trend was recorded in 0.2 g L^−1*^ “Red sonnet.” Projected and total surface root area were highest in “Red sonnet” plants at 0.2 g L^−1^ rate (respectively 232.1 and 720.1 cm^2^).

The interaction D ^*^ M ^*^ CV was found to be significant for all parameters (Table S5, Supplementary materials).

### Nitrogen content

The animal-derived PH biostimulant had a significant effect on total leaf and root nitrogen content in the snapdragon (Table 6). Applying the biostimulant improved the nitrogen plant content compared to the control, although no significant differences were found comparing the effects of the two doses of biostimulant applied (foliar-N +16% and root-N +8%). Compared to spraying, drenching led to higher nitrogen values (both foliar-N and root-N +3%). The cultivar factor had a significant effect on plant nitrogen content, where “Red sonnet” recorded the highest values (foliar-N: 406.5 mg kg^−1^ and root-N: 295.8 mg kg^−1^).

**Table 6:** Main effects of biostimulant dose, application method and cultivar on leaf and root total N content of snapdragon plants.

**Treatments**	**Total N (mg kg^−1^)**	
	**Leaf**	**Root**
**DOSE (g L^−1^) (D)**		
0	361.1b	277.5b
0.1	422.5a	300.6a
0.2	417.8a	299.1a
**METHOD (M)**		
Foliar spray	389.3b	281.4b
Root drenching	401.9a	290.8a
**CULTIVAR (CV)**		
Yellow floral showers	395.7b	288.9b
Red sonnet	406.5a	295.8a
**Significance**		
D	^***^	^***^
M	^***^	^***^
CV	^*^	^**^
D × M	^*^	ns
D × CV	^*^	ns
M × CV	ns	ns
D × M × CV	ns	ns

*Different letters within each column indicate significant differences according to Duncan's multiple-range test (P ≤ 0.05); ns, non significant*,

* *P < 0.05*,

** 0.01, and

*** *0.001, indicate level of significance*.

Both the interactions D ^*^ M and D ^*^ CV were found to be significant only for total leaf-N content (Tables S1, S6, Supplementary materials).

### Leaf gas exchange and chlorophylls fluorescence

The biostimulant use in snapdragon had a positive influence on the parameters related to the leaf gas exchange (Table 7). Irrespectively of the two doses applied, the biostimulant significantly influenced the leaf net photosynthesis (+52%), transpiration rate (+55%), stomatal conductance (0.8%), and concentration of CO_2_ in intracellular spaces (+9%), compared to control plants. On the other hand, the electronic transport was significantly higher at 0.1 g L^−1^ of animal-derived biostimulant (+129.1) compared to the other treatments.

**Table 7:** Main effects of biostimulant dose, application method and cultivar on net photosynthesis, transpiration rate, stomatal conductance, concentration of CO_2_, electronic transport rate, efficiency of Photosystem II and fluorescence parameters in snapdragon plants.

**Treatments**	**Net photosynthesis (μmol CO_2_ m^−2^ s^−1^)**	**Transpiration rate (mmol H_2_O m^−2^ s^−1^)**	**Stomatal conductance (mmol H_2_O m^−2^ s^−1^)**	**Concentration of CO_2_ (ppm)**	**Electronic transport rate (μmol e^−^ m^−2^ s^−1^)**	**Efficiency of photosystem II (F'v/F'm)**	**Chlorophylls fluorescence (Fv/Fm)**
**DOSE (g L^−1^) (D)**							
0	9.43 b	1.83b	0.08b	175.8b	112.1c	0.26b	0.83a
0.1	14.51a	2.88a	0.15a	198.9a	129.1a	0.29a	0.84a
0.2	14.21a	2.82a	0.14a	209.1a	120.7b	0.27b	0.84a
**METHOD (M)**							
Foliar spray	11.96a	2.28b	0.10b	184.6b	117.7a	0.26b	0.84a
Root drenching	12.85a	2.75a	0.14a	204.6a	124.0a	0.29a	0.84a
**CULTIVAR (CV)**							
Yellow floral showers	12.18a	2.82a	0.13a	205.8a	132.9a	0.30a	0.81b
Red sonnet	12.62a	2.19b	0.11a	183.3b	108.8b	0.25b	0.87a
**SIGNIFICANCE**							
D	^***^	^*^	^**^	^**^	^*^	^**^	ns
M	ns	^*^	^*^	^**^	ns	^**^	ns
CV	ns	^*^	ns	ns	^*^	^*^	^*^
D × M	ns	ns	ns	^*^	ns	^**^	ns
D × CV	ns	^*^	ns	^**^	ns	^*^	ns
M × CV	ns	ns	ns	^*^	ns	^*^	ns
D × M × CV	ns	ns	ns	ns	ns	^**^	ns

*Different letters within each column indicate significant differences according to Duncan's multiple-range test (P ≤ 0.05)*.

*ns, non significant*,

* *P < 0.05*,

** 0.01, and

*** *0.001, indicate level of significance*.

The biostimulant method did not affect the plant photosynthetic rate, however it had a significant effect on the transpiration rate (+21%), stomatal conductance (+40%), and concentration of CO_2_ in intracellular spaces (+11%), where the highest values occurred using the root application method. With reference to leaf gas exchange parameters, “Yellow floral showers” had significantly higher values in terms of concentration of CO_2_ in intracellular spaces, transpiration rate, and electronic transport, whereas a higher water use efficiency was found in “Red sonnet.”

The interactions between the dose and application method (Table S1, Supplementary materials) and between method ^*^ cultivar (Table S4, Supplementary materials) were significant only for the CO_2_ concentration in the intracellular spaces. In addition, the interaction between the dose and cultivar for transpiration rate and CO_2_ concentration were significant only in the intracellular spaces (Table S6, Supplementary materials).

The effects of the biostimulant on the fluorescence parameters of snapdragon are shown in Table 7. The application of a biostimulant dose of 0.1 g L^−1^ resulted in a significant increase in the efficiency of Photosystem II compared to the other treatments. The values of this parameter increased significantly when the biostimulant was applied through root application (+11%). In addition, the efficiency of Photosystem II was significantly higher in the “Yellow floral showers” compared to “Red sonnet.” A highly significant interaction was found between the biostimulant dose and application method (Table 7) as well as among the dose, application method and cultivar (Table S2, Supplementary materials). No difference was found when applying biostimulant in relation to the chlorophylls fluorescence, with the exception of the cultivar factor where chlorophylls fluorescence was higher in “Red sonnet.”

## Discussion

To the best of our knowledge, the effects of using an animal-derived PH biostimulant on the growth parameters and physiological behavior of potted snapdragon plants have not previously been reported. Therefore, the cross-referencing in the discussion of the findings in this study will be based on the results available from other plant species.

Biostimulants have been found to increase the growth traits in many horticultural crops, in terms of increased shoot, root biomass, nutrient uptake, and plant yield (Ertani et al., 2009; Kunicki et al., 2010; Colla et al., 2014, 2015; Santi et al., 2017). In the present study, the application of an animal-derived biostimulant resulted in an improvement in morphological and qualitative traits (Table 2). Ertani et al. (2009) reported an increase in root and leaf growth in maize treated with an animal-derived biostimulant, which also induced morphological changes in the root system increasing the root dry weight of plants (Table 3). In agreement with our findings, the same authors found that the most evident plant increments were observed when the biostimulant was applied in the range of 0.01–0.1 g L^−1^. In another study, Quartieri et al. (2002) tested the effect of an animal-derived biostimulant as a foliar application in potted kiwifruit plants and found an improvement in hypogeal plant dry weight. In nursery-grown passionfruit, Morales-Pajan and Stall (2004) observed that foliar applications with an animal-derived biostimulant increased the seedling growth.

Regarding ornamental flower crops, the present work is in line with (Tables 2, 4, 5) reports by De Lucia and Vecchietti (2012), who investigated the effects of three different agricultural biostimulants based on hydrolyzed proteins from algae, animal derived-protein hydrolysate and alfalfa origin on L.A. lily hybrids (*Lilium longiflorum* x *L. asiaticum*) grown in a soilless system. The three biostimulants, applied as foliar spray or soil drenching, led to similar performances, reducing the crop cycle of plants and increasing the leaf area and flower buds; the plant root system was also more developed compared to the control.

Applying an animal-derived biostimulant to tomato grown under greenhouse conditions, led to an increase in plant height and number of flowers per plant compared to untreated plants (Parrado et al., 2008). Botta (2013) conducted a cold stress trial on lettuce under controlled environmental conditions using an animal-derived biostimulant. They found that the biostimulant application led to higher shoot and root fresh weights and stomatal conductance compared to untreated control-plants.

When investigating the effects of fish-derived biostimulants on the growth of lettuce, Xu and Mou (2017) found that biostimulants significantly increased the lettuce leaf number per plant, shoot and root dry weight, but had no effect on leaf area. In contrast to the general trend of the results available, studying spinach plants from different cultivars, Kunicki et al. (2010) observed that applying an animal-derived biostimulant as a foliar method had no effect on plant yield, however the cultivar factor significantly influenced the spinach dry weight. Ruiz et al. (2000) reported that the foliar application of an animal-derived biostimulant reduced growth and yield as well as the root nitrate uptake and nitrogen efficiency in pepper.

Our results (Table 6), instead, suggest that snapdragon plants treated with a biostimulant by drenching, increased foliar and root nitrogen content, although no differences were found comparing the two doses applied. Root length and surface area are an integrative indicator of the plant response to water and nutrient uptake (Clothier and Green, 1997; Ryser, 2006). Colla et al. (2014) showed that in tomato the increase in root apparatus resulting from protein hydrolysate applications may also have contributed to increasing the nitrogen uptake by plants.

Other authors have highlighted the positive effects of biostimulants in plant nutrition. Studying cucumber development, Rauthan and Schnitzer (1981) reported the growth of above and below ground plant parts. In addition the use of a biostimulant on bermudagrass was found to enhance the root surface area (Tucker et al., 2006). In agreement with previous studies, it was also found that animal-derived biostimulants stimulate N metabolism and assimilation (Baglieri et al., 2014; Calvo et al., 2014; Colla et al., 2015; Rouphael et al., 2017b). In a review of the literature, Maini (2006) reported that the components of an animal-derived biostimulant preparation penetrated rapidly into treated leaves, and were subsequently distributed to other leaves. Schiavon et al. (2008) also reported that the enzyme activity in N reduction and assimilation was stimulated by an animal-derived biostimulant applied to maize plants.

In agreement with our findings, Halpern et al. (2015) and Santi et al. (2017) also demonstrated the positive effects of biostimulant application on plant nutrient uptake including nitrogen. In the present study, no case of snapdragon plant death was observed, thus demonstrating that the application of the animal-derived biostimulant maintained crop uniformity, which was also demonstrated by Tsouvaltzis et al. (2014) in greenhouse lettuce crops.

According to findings by Ferrini and Nicese (2002) for English oak and by Xu and Mou (2017) for lettuce, our use of a plant biostimulant enhanced many physiological parameters such as photosynthetic rate, stomatal conductance and transpiration rate, thus ensuring a higher carbon assimilation efficiency (Table 7).

As suggested by Yakhin et al. (2017), amino acid based biostimulants are readily absorbed and translocated by plant tissues. They also function as modulators of stomatal opening once absorbed, acting on the stimulation of photosynthesis or down regulating the plant stress signaling pathway. A high photosynthetic rate of shoots secures high root activity by supplying a sufficient amount of photosynthates to the roots (Yang et al., 2004). The biostimulant used in this study did not alter chlorophyll fluorescence (Table 7) as also found by Xu and Mou (2017) in lettuce, suggesting that there were no stress-induced perturbations in the photosynthetic apparatus (Baker and Rosenqvist, 2004).

## Conclusions

Based on our data, the biostimulant enhanced the ornamental quality in potted snapdragon. Plant morphological and qualitative traits, leaf and root-N content, photosynthetic rate, transpiration rate, and stomatal conductance were significantly increased by the biostimulant application compared to the control, regardless of the dose. The lowest dose also resulted in the best effect on both the dry weight of above-ground plant and the root system.

The root drenching method enhanced the plant shoot number, ground dry weight, root morphology, leaf, and root-N content and gas exchange. A higher pot quality was obtained in “Red sonnet” compared to “Yellow floral shower.”

Based on these findings, applying the biostimulant at the lowest dose to potted snapdragon, as part of a fertilizing regime, improves the crop quality in an agro-environmental sustainable way.

## Author contributions

GC carried out the experiment, processed the experimental data, performed the analysis, designed the figures, and wrote part of the research dealing with animal-derived PHs biostimulant effects on plant growth; EP conducted the physiological measurements and wrote part of the research dealing with animal-derived PHs biostimulant effects on gas exchange and fluorescence; GCo gave support in the data analysis and interpretation; VT provided background information on PHs based biostimulants; BD developed the concept of this experiment, designed the study, wrote part of the research dealing with animal-derived PHs biostimulant effects on root system. All authors provided critical feedback, made contributions to analysis and interpretation of data, discussed the results, contributed to the writing of the manuscript and gave final approval of the version to be published.

### Conflict of interest statement

The authors declare that the research was conducted in the absence of any commercial or financial relationships that could be construed as a potential conflict of interest. The reviewer CC and the handling editor declared their shared affiliation.

## Supplementary material

Table S1 The effects of the interaction between biostimulant dose and application methods on snapdragon plants characteristics: shoots (n /plant), flower (n /plant), flower dry weight (g/ plant), ground dry weight (g/plant), projected root area (cm^2^ /plant), total leaf-N (mg kg^−1^), concentration of CO_2_ (ppm).

Table S2 The effects of the interaction between biostimulant dose application methods and cultivar on snapdragon plants characteristics: total shoot length (cm /plant), total leaf area (cm^2^/plant), flower dry weight (g/ plant), total above dry weight (g/plant), projected root area (cm^2^/plant), and efficiency of Photosystem II (F'v/F'm).

Table S3 The effects of the interaction between biostimulant dose and cultivar on snapdragon plants characteristics: shoots (n /plant), flower (n /plant), leaf (n/plant), flower dry weight (g/ plant), total above dry weight (g/plant), ground dry weight (g/plant).

Table S4 The effects of the interaction between biostimulant application methods and cultivar on snapdragon plants characteristics: leaf (n/plant), ground dry weight (g/plant), projected root area (cm^2^ /plant), and concentration of CO_2_ (ppm).

Table S5 The effects of the interaction between biostimulant dose application methods and cultivar on snapdragon plants characteristics: total root length, root diameter, root volume, root tips, root crossings, and root forks.

Table S6 The effects of the interaction between biostimulant dose and cultivar on snapdragon plants characteristics: projected root area (cm_2_ /plant), total leaf-N content (mg kg^−1^), transpiration rate (mmol H_2_O m^−2^ s^−1^), and concentration of CO_2_ (ppm).
