# Supplementary material for: Effects of an Animal-Derived Biostimulant on the Growth and Physiological Parameters of Potted Snapdragon (Antirrhinum majus L.)
Source: Front Plant Sci. 2018 Jun 20;9:861. doi: 10.3389/fpls.2018.00861 (PMC6019948; doi:10.3389/fpls.2018.00861)
Supplement: Table S1 — The effects of the interaction between biostimulant dose and application methods on snapdragon plants characteristics: shoots (n /plant), flower (n /plant), flower dry weight (g/ plant), ground dry weight (g/plant), projected root area (cm2 /plant), total leaf-N (mg kg−1), concentration of CO2 (ppm). [file Table_1.DOCX]

| Treatments | Shoots (n /plant) | | Flower (n /plant) | | Flower dry weight  (g/ plant) | | Ground dry weight  (g/ plant) | | Projected root area  (cm^2^ /plant) | | Total  leaf-N (mg kg^-1^) | | Concentration of CO_2_  (ppm) | |
| --- | --- | --- | --- | --- | --- | --- | --- | --- | --- | --- | --- | --- | --- | --- |
|  |  | | | | | | | | | | | | | |
| Dose (g L^-1^) | Method | | | | | | | | | | | | | |
|  | Foliar  spray | Root drenching | Foliar  spray | Root drenching | Foliar  spray | Root drenching | Foliar  spray | Root drenching | Foliar  spray | Root drenching | Foliar  spray | Root drenching | Foliar  spray | Root drenching |
| 0 | 5.33b | 5.31b | 59.6c | 59.3c | 2.70b | 2.67b | 2.02b | 2.02b | 132.4d | 140.3d | 361.3c | 369.8c | 175.8b | 174.3b |
| 0.1 | 6.17ab | 6.83a | 96.7a | 82.8b | 4.79a | 2.87b | 2.73ab | 3.20a | 193.4bc | 248.7a | 422.0a | 423.8a | 176.3b | 221.6a |
| 0.2 | 5.67ab | 6.79a | 102.2a | 98.7a | 3.97ab | 3.46ab | 2.27b | 2.80ab | 147.9cd | 196.8b | 407.7b | 428.0a | 201.7ab | 216.4a |

Table S1 - The effects of the interaction between biostimulant dose and application methods on snapdragon plants characteristics: shoots (n /plant), flower (n /plant), flower dry weight (g/ plant), ground dry weight (g/plant), projected root area (cm^2^ /plant), total leaf-N (mg kg^-1^), concentration of CO_2_  (ppm).

Mean sharing different letters in each trait differs significantly at P ≤ 0.05
