# Supplementary material for: Effects of an Animal-Derived Biostimulant on the Growth and Physiological Parameters of Potted Snapdragon (Antirrhinum majus L.)
Source: Front Plant Sci. 2018 Jun 20;9:861. doi: 10.3389/fpls.2018.00861 (PMC6019948; doi:10.3389/fpls.2018.00861)
Supplement: Table S2 — The effects of the interaction between biostimulant dose application methods and cultivar on snapdragon plants characteristics: total shoot length (cm /plant), total leaf area (cm2/plant), flower dry weight (g/ plant), total above dry weight (g/plant), projected root area (cm2/plant), and efficiency of Photosystem II (F'v/F'm). [file Table_2.DOCX]

Table S2 - The effects of the interaction between biostimulant dose application methods and cultivar on snapdragon plants characteristics: total shoot length (cm /plant), total leaf area (cm2 /plant), flower dry weight (g/ plant), total above dry weight (g/plant), projected root area (cm2 /plant) and efficiency of Photosystem II (F'v/F'm).

| Treatments | | Total shoot length  (cm/plant) | | Total leaf area  (cm^2^ /plant) | | Flower dry weight  (g/ plant) | | Total above-ground dry weight  (g/ plant) | | Projected root area  (cm^2^ /plant) | | Efficiency of Photosystem II (F'v/F'm) | |
| --- | --- | --- | --- | --- | --- | --- | --- | --- | --- | --- | --- | --- | --- |
|  |  | Cultivar | | Cultivar | | Cultivar | | Cultivar | | Cultivar | | Cultivar | |
| Dose  (g L^-1^) | Method | Yellow  floral showers | Red  sonnet | Yellow  floral  showers | Red  sonnet | Yellow  floral  showers | Red  sonnet | Yellow  floral showers | Red  sonnet | Yellow  floral showers | Red  sonnet | Yellow  floral showers | Red  sonnet |
| 0 | Foliar spray | 81.6e | 255.8bc | 428.5d | 1002.8bc | 1.72d | 3.58c | 16.7d | 50.2a | 93.2f | 171.7cd | 0.28abc | 0.25bc |
|  | Root drenching | 77.3e | 250.1bc | 412.9d | 987.6bc | 1.70d | 3.61c | 16.54d | 47.3a | 98.1f | 182.4c | 0.28abc | 0.25bc |
| 0.1 | Foliar spray | 99.3de | 295.8a | 556.2d | 938.9c | 3.77c | 5.81a | 29.3c | 50.2a | 196.0c | 190.7c | 0.35a | 0.23c |
|  | Root drenching | 104.0de | 263.3bc | 472.7d | 989.5bc | 2.22d | 3.51c | 28.3c | 46.0ab | 225.5b | 271.9a | 0.31abc | 0.28abc |
| 0.2 | Foliar spray | 88.7e | 278.7ab | 538.3d | 1138.9a | 1.99d | 5.95a | 26.9c | 45.3b | 124.7e | 172.1cd | 0.27abc | 0.23c |
|  | Root drenching | 118.7d | 247.3c | 554.9d | 1091b | 2.24d | 4.69b | 28.4c | 46.3ab | 151.7d | 242.0b | 0.32ab | 0.27abc |

Mean sharing different letters in each trait differs significantly at P ≤ 0.05
