# Supplementary material for: Effects of an Animal-Derived Biostimulant on the Growth and Physiological Parameters of Potted Snapdragon (Antirrhinum majus L.)
Source: Front Plant Sci. 2018 Jun 20;9:861. doi: 10.3389/fpls.2018.00861 (PMC6019948; doi:10.3389/fpls.2018.00861)
Supplement: Table S3 — The effects of the interaction between biostimulant dose and cultivar on snapdragon plants characteristics: shoots (n /plant), flower (n /plant), leaf (n/plant), flower dry weight (g/ plant), total above dry weight (g/plant), ground dry weight (g/plant). [file Table_3.DOCX]

Table S3 - The effects of the interaction between biostimulant dose and cultivar on snapdragon plants characteristics: shoots (n /plant), flower (n /plant), leaf (n/plant), flower dry weight (g/ plant), total above-ground dry weight (g/plant), ground dry weight (g/plant).

| Treatments | Shoots (n /plant) | | Flower (n /plant) | | Leaf (n/plant) | | Flower  dry weight  (g/plant) | | Total above  dry weight  (g/plant) | | Ground  dry weight  (g/plant) | |
| --- | --- | --- | --- | --- | --- | --- | --- | --- | --- | --- | --- | --- |
|  | Cultivar (CV) | | | | | | | | | | | |
|  | Yellow  floral showers | Red  sonnet | Yellow  floral showers | Red  sonnet | Yellow  floral showers | Red  sonnet | Yellow  Floral  showers | Red  sonnet | Yellow  floral  showers | Red  sonnet | Yellow  floral  showers | Red  sonnet |
| Dose (D)  (g L^-1^) |  |  |  |  |  |  |  |  |  |  |  |  |
| 0 | 5.30b | 5.29b | 36.5e | 82.7c | 334.8d | 666.2b | 1.71d | 3.68b | 16.73d | 50.17a | 1.37e | 2.67bc |
| 0.1 | 5.17b | 7.67a | 66.3d | 113.2b | 471.2c | 860.1a | 2.99bc | 4.66a | 28.82c | 48.13ab | 2.50c | 3.43a |
| 0.2 | 5.33b | 7.17a | 59.0d | 141.8a | 442.5c | 869.8a | 2.12cd | 5.32a | 27.63c | 45.82b | 2.13d | 2.93b |

Mean sharing different letters in each trait differs significantly at P ≤ 0.05
