# Supplementary material for: Effects of an Animal-Derived Biostimulant on the Growth and Physiological Parameters of Potted Snapdragon (Antirrhinum majus L.)
Source: Front Plant Sci. 2018 Jun 20;9:861. doi: 10.3389/fpls.2018.00861 (PMC6019948; doi:10.3389/fpls.2018.00861)
Supplement: Table S4 — The effects of the interaction between biostimulant application methods and cultivar on snapdragon plants characteristics: leaf (n/plant), ground dry weight (g/plant), projected root area (cm2 /plant), and concentration of CO2 (ppm). [file Table_4.DOCX]

Table S4 - The effects of the interaction between biostimulant application methods and cultivar on snapdragon plants characteristics: leaf (n/plant), ground dry weight (g/plant), projected root area (cm^2^ /plant) and concentration of CO_2_  (ppm).

| Treatments | Leaf  (n/plant) | | Ground dry weight  (g/ plant) | | Projected root area  (cm^2^ /plant) | | Concentration  of CO_2_ (ppm) | |
| --- | --- | --- | --- | --- | --- | --- | --- | --- |
|  | Method (M) | | | | | | | |
| Cultivar (CV) | Foliar spray | Root drenching | Foliar  spray | Root drenching | Foliar  spray | Root drenching | Foliar spray | Root drenching |
| Yellow floral showers | 421.6b | 762.7a | 1.90b | 2.78a | 138.0b | 158.4b | 188.2b | 223.5a |
| Red sonnet | 410.7b | 841.4a | 2.10b | 3.24a | 177.8b | 232.1a | 181.0b | 185.7b |

Mean sharing different letters in each trait differs significantly at P ≤ 0.05
