# Supplementary material for: Effects of an Animal-Derived Biostimulant on the Growth and Physiological Parameters of Potted Snapdragon (Antirrhinum majus L.)
Source: Front Plant Sci. 2018 Jun 20;9:861. doi: 10.3389/fpls.2018.00861 (PMC6019948; doi:10.3389/fpls.2018.00861)
Supplement: Table S5 — The effects of the interaction between biostimulant dose application methods and cultivar on snapdragon plants characteristics: total root length, root diameter, root volume, root tips, root crossings, and root forks. [file Table_5.DOCX]

Table S5 - The effects of the interaction between biostimulant dose application methods and cultivar on snapdragon plants characteristics: total root length, root diameter, root volume, root tips, root crossings and root forks.

| Treatments | | Total root length (m 10^3^/plant) | | Root diameter  (mm) | | Root volume  (cm^3^/plant) | | Root tips  (n 10^3^/plant) | | Root crossings  (n 10^3^/plant) | | Root forks  (n 10^3^/plant) | |
| --- | --- | --- | --- | --- | --- | --- | --- | --- | --- | --- | --- | --- | --- |
|  |  | Cultivar | | Cultivar | | Cultivar | | Cultivar | | Cultivar | | Cultivar | |
| Dose  (g L^-1^) | Method | Yellow  floral showers | Red  sonnet | Yellow  floral  showers | Red  sonnet | Yellow  floral  showers | Red  sonnet | Yellow  floral showers | Red  sonnet | Yellow  floral showers | Red  sonnet | Yellow  floral showers | Red  sonnet |
| 0 | Foliar spray | 1.86h | 4.58e | 1.01d | 1.02d | 3.77i | 5.23g | 12.3k | 30.0f | 2.05k | 7.52f | 15.9k | 39.3g |
|  | Root drenching | 1.94h | 4.80d | 1.07d | 1.06d | 4.02i | 5.42g | 12.9j | 31.1d | 2.14k | 7.86e | 16.8j | 41.1e |
| 0.1 | Foliar spray | 4.45e | 4.56e | 1.34bc | 1.25c | 6.85d | 6.36e | 26.1g | 35.8b | 6.94g | 6.47h | 41.2e | 39.9f |
|  | Root drenching | 5.10c | 6.21a | 1.36b | 1.74a | 7.87c | 9.53a | 23.9i | 42.6a | 8.89c | 10.2a | 49.1c | 59.7a |
| 0.2 | Foliar spray | 3.11g | 4.01f | 0.79e | 0.87e | 3.79i | 5.77f | 25.6h | 30.7e | 5.51j | 8.02d | 30.6i | 43.2d |
|  | Root drenching | 4.05f | 5.53b | 1.12d | 1.33bc | 4.64h | 8.47b | 35.9b | 31.5c | 5.93i | 9.75b | 34.4h | 57.0b |

Mean sharing different letters in each trait differs significantly at P ≤ 0.05
