# Supplementary material for: Effects of an Animal-Derived Biostimulant on the Growth and Physiological Parameters of Potted Snapdragon (Antirrhinum majus L.)
Source: Front Plant Sci. 2018 Jun 20;9:861. doi: 10.3389/fpls.2018.00861 (PMC6019948; doi:10.3389/fpls.2018.00861)
Supplement: Table S6 — The effects of the interaction between biostimulant dose and cultivar on snapdragon plants characteristics: projected root area (cm2 /plant), total leaf-N content (mg kg−1), transpiration rate (mmol H2O m−2 s−1), and concentration of CO2 (ppm). [file Table_6.DOCX]

Table S6 - The effects of the interaction between biostimulant dose and cultivar on snapdragon plants characteristics: projected root area (cm^2^ /plant),

total leaf – N content (mg kg ^-1^), transpiration rate (mmol H_2_O m^-2^ s^-1^ ) and concentration of CO_2_  (ppm).

| Treatments | Projected root area  (cm^2^ /plant) | | Total  leaf-N (mg kg^-1^) | | Transpiration rate  (mmol H_2_O m^-2^ s^-1^) | | Concentration of CO_2_ (ppm) | |
| --- | --- | --- | --- | --- | --- | --- | --- | --- |
|  | Cultivar (CV) | | | | | | | |
|  | Yellow  floral showers | Red  sonnet | Yellow  floral showers | Red  sonnet | Yellow  floral showers | Red  sonnet | Yellow  floral showers | Red  sonnet |
| Dose (D)  (g L^-1^) |  |  |  |  |  |  |  |  |
| 0 | 95.7d | 177.1b | 358.2d | 373.0c | 1.49b | 2.18b | 168.1b | 183.6b |
| 0.1 | 210.8ab | 231.3a | 421.7a | 424.2a | 3.56a | 2.21b | 217.8a | 180.1b |
| 0.2 | 138.2c | 206.5ab | 410.7b | 425.0a | 3.43a | 2.21b | 231.7a | 186.4b |

Mean sharing different letters in each trait differs significantly at P ≤ 0.05
